# Jamming transitions in cancer

**DOI:** 10.1088/1361-6463/aa8e83

**Published:** 2017-10-27

**Authors:** Linda Oswald, Steffen Grosser, David M Smith, Josef A Käs

**Affiliations:** 1 University of Leipzig, Faculty of Physics and Earth Sciences, Debye Institute, Linnéstr. 5, 04103 Leipzig, Germany; 2 Fraunhofer Institute for Cell Therapy and Immunology, Perlickstr. 1, 04103 Leipzig, Germany

**Keywords:** jamming, cell mechanics, epithelial, EMT, cell migration, cancer, metastasis

## Abstract

The traditional picture of tissues, where they are treated as liquids defined by properties such as surface tension or viscosity has been redefined during the last few decades by the more fundamental question: under which conditions do tissues display liquid-like or solid-like behaviour? As a result, basic concepts arising from the treatment of tissues as solid matter, such as cellular jamming and glassy tissues, have shifted into the current focus of biophysical research. Here, we review recent works examining the phase states of tissue with an emphasis on jamming transitions in cancer. When metastasis occurs, cells gain the ability to leave the primary tumour and infiltrate other parts of the body. Recent studies have shown that a linkage between an unjamming transition and tumour progression indeed exists, which could be of importance when designing surgery and treatment approaches for cancer patients.

## 1 Introduction

### 1.1 Significance of cellular mechanics for cancer

Cancer is one of the most common causes of death in the world. It is characterised by uncontrolled cell growth, invasion into surrounding tissues and the eventual appereance of metastases in other parts of the body [1]. According to the World Health Organisation, 14 million new cases of cancer and 8.2 million deaths due to cancer were reported worldwide in 2012 [2].

Finding a broadly applicable treatment strategy for cancer is challenging since it can manifest itself in an abundant number of forms. Many therapies are highly specific and only consider the biochemical aspects of the disease. One such example is a targeted therapy based on the growth factor and receptor HER2, which is effective for only about 20% of breast cancer patients [3]. However, to develop a therapeutic approach which is broadly applicable for many types of cancer, it may be expedient to consider not only molecular details, but also coarse-grained variables such as mechanical tissue properties.

Cancer is a collective phenomenon; since single malignant cancer cells can often be easily handled by the immune system, metastasis is rather inefficient. The vast majority of cells are eliminated on the way to distant tissues [4, 5]. Although most biological approaches aim to understand and fight the disease on a single-cell level it is nevertheless important to investigate how the cancer cell ensemble within the primary tumour is stably maintained until metastasis occurs, enabling cells to leave the tumour and infiltrate other parts of the body. These metastatic cells seem to be hindered by two mechanical mechanisms: blocking and jamming [6, 7].

*Blocking* refers to an external steric barrier which cells cannot pass, while *jamming* describes a rigidity transition caused by mutual steric hindrance of the tumour cells. Cellular jamming differs from jamming in inert matter in two aspects: first, cells are active particles, and second, the jamming transition occurs at a packing density of one, i.e. for a 2D system at confluence. In this article, we will review research on the phase state of cellular systems pointing towards the importance of cell jamming and its linkage to cancer progression.

**Figure 1:**
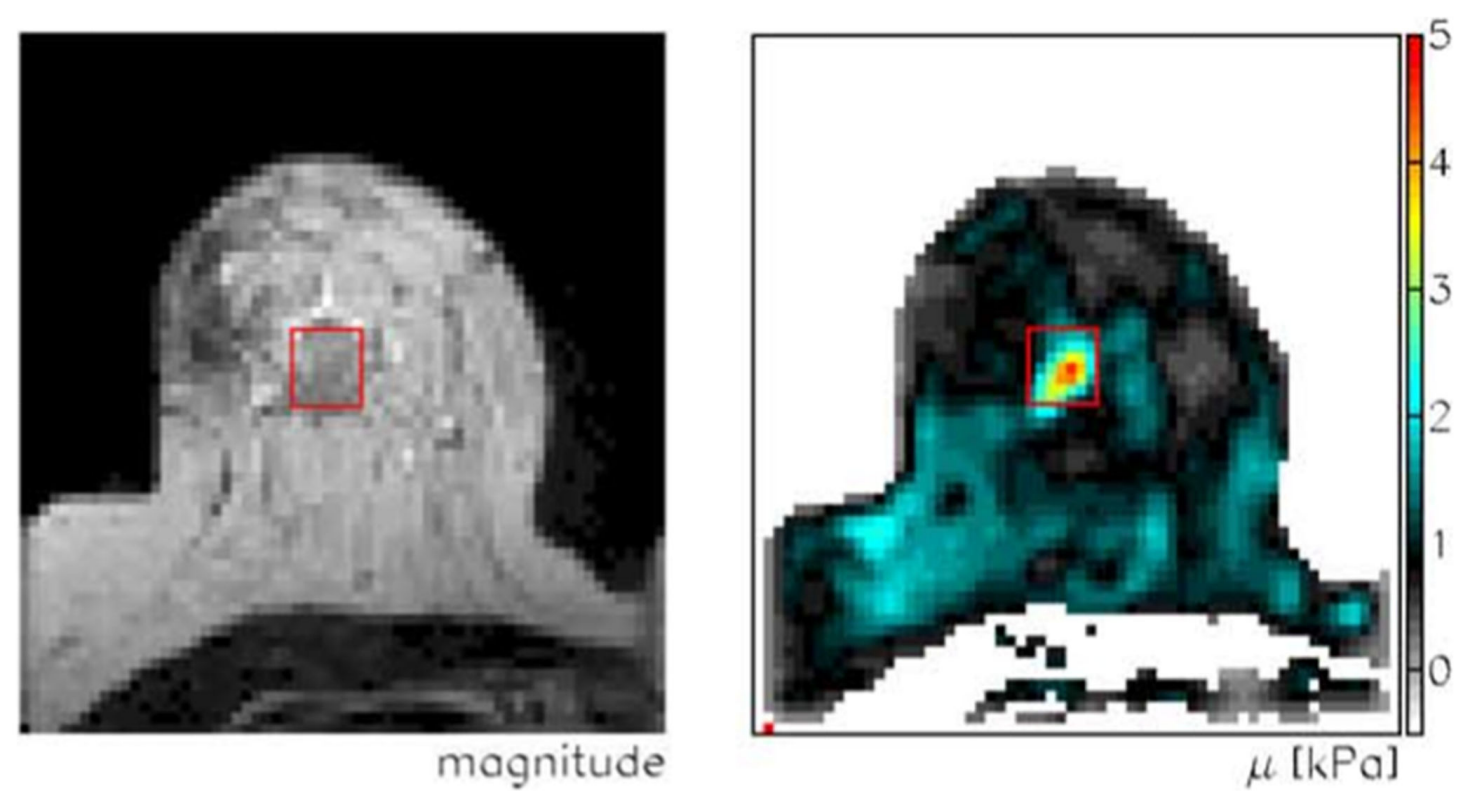
Magnetic resonance imaging (left) and elastography (right) of a breast tumour. The tumour (red rectangle) is stiffer than its surroundings. Reprinted from [8], Copyright 2005, with permission from Elsevier.

In the last few decades, there has been growing evidence that cell mechanics, including viscoelasticity, adhesivity and active soft matter behaviour, play an important role in not only tissue formation and maintenance, but also in disease progression. It is long known that tumours often appear stiffer than their surroundings, enabling physicians to detect cancer as lumps in the body. Additionally, modern non-invasive probing techniques such as magnetic resonance elastography show that many tumours are stiffer than surrounding tissue (see figure 1) [8]. Mechanosensing, the ability of cells to sense external mechanical stimuli such as compression, shear stress and substrate stiffness, can affect cell division rates, cell–cell interactions, cell-matrix interactions and cell movement [9].

**Figure 2:**
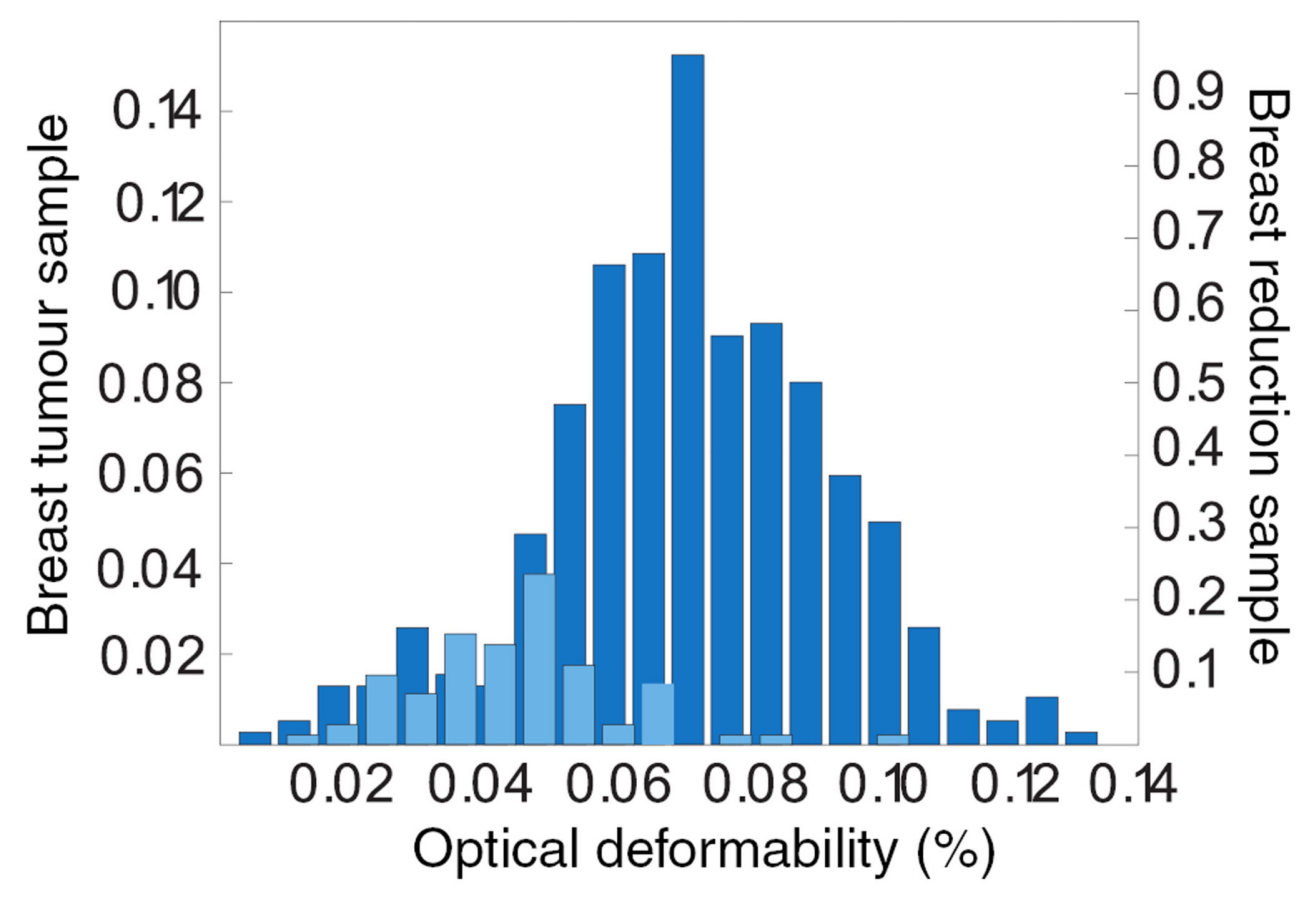
Single-cell deformability in the *Optical Stretcher* for primary tissue samples. Tumour tissue (dark blue) contains a higher fraction of softer cells than normal tissue (light blue). Reprinted by permission from Macmillan Publishers Ltd: Nature Physics [11], Copyright 2010.

Another example for the importance of mechanics is the cytoskeleton. The cytoskeleton is a dynamic biopolymer scaffold which is involved in many cellular processes. Although it is influenced by a large number of biochemical cues, it is also the dominant mechanical object within the cell, providing both stability as well as the basis for cell movement [10]. In certain cancers as well as in well-characterised cancer cell lines, cells are softer, i.e. more deformable, than non-cancerous cells (see figure 2) [11–15], possibly due to a down-regulation of cytoskeletal actin.

Softening of cells is reported to be beneficial for invasion [16, 17], however, this exposes an apparent discrepancy in the picture, whereby stiff tumours contain softer cells (see figures 1 and 2). Such a contradiction between single-cell and tissue-level properties might be resolved when considering emergent phenomena, such as jamming transitions, which will be discussed in more detail below.

**Figure 3:**
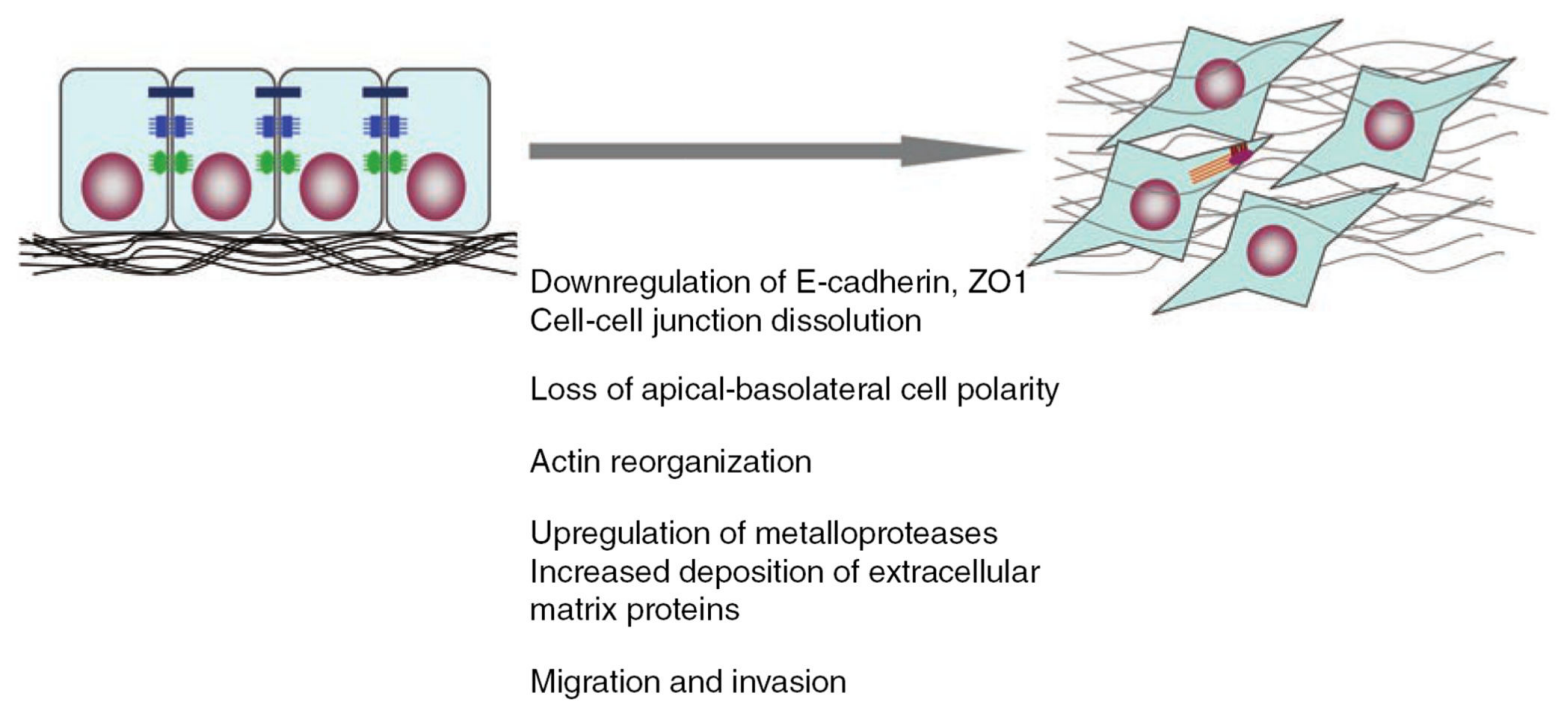
Schematic epithelial mesenchymal transition (EMT) from polarised epithelial cells (left) to mesenchymal cells anchored in the ECM (right). Reprinted by permission from Macmillan Publishers Ltd: Cell Research [19], Copyright 2009.

A process that is often associated with cancer and changes cell properties is the epithelial–mesenchymal transition (EMT). Its main feature is a transformation of phenotype (see figure 3) from polarised epithelial cells to elongated mesenchymal cells which are anchored in the extracellular matrix (ECM) [18].

EMT is characterised by enhanced migration and invasiveness, altered production of ECM, decreased cell–cell adhesion and degradation of the basement membrane [18, 20]. E-cadherins are downregulated while N- and P-cadherins are upregulated [21–23]. A more comprehensive list of EMT markers can be found in [21].

EMT is reported to play a role in tissue repair, inflammation and tumour progression [18], however, its role in cancer is still debated since it is neither a necessary nor a sufficient marker for malignant behaviour [21]. Nevertheless, the presence of EMT markers are associated with poor clinical outcome for certain cancer types, such as colorectal, breast and ovarian cancer [21, 24]. For these and other carcinomas, i.e. epithelial cancers, EMT is proposed to be a critical process in tumour progression and particularly, in metastasis [22].

Since EMT is often concurrent with a transition from collective movement to single cell movement, a change in cell–cell adhesion and a change of cell contractility induced by a remodelled actin cytoskeleton [22], it has a significant influence on mechanical cell properties.

Consequently, EMT is likely to be an important corner-stone for understanding the physics of cancer [18, 22, 25].

### 1.2 Jamming and glass transition

Glasses are amorphous solids lacking the long-range order of crystalline solids. The transition from a liquid to a glass is reversible and temperature-induced, where the cooling speed is a critical factor [26]. After a supercooling process, particles are locked into a non-equilibrium configuration and the structural relaxation time increases significantly [27].

A very similar transition is the jamming transition accompanied by an abrupt dynamical arrest [28]. It can be induced by increasing density/volume fraction, thereby hindering the particles from exploring the whole phase space [29]. Macroscopic examples for jammed ‘materials’ are sandpiles, traffic jams and foams [30].

**Figure 4:**
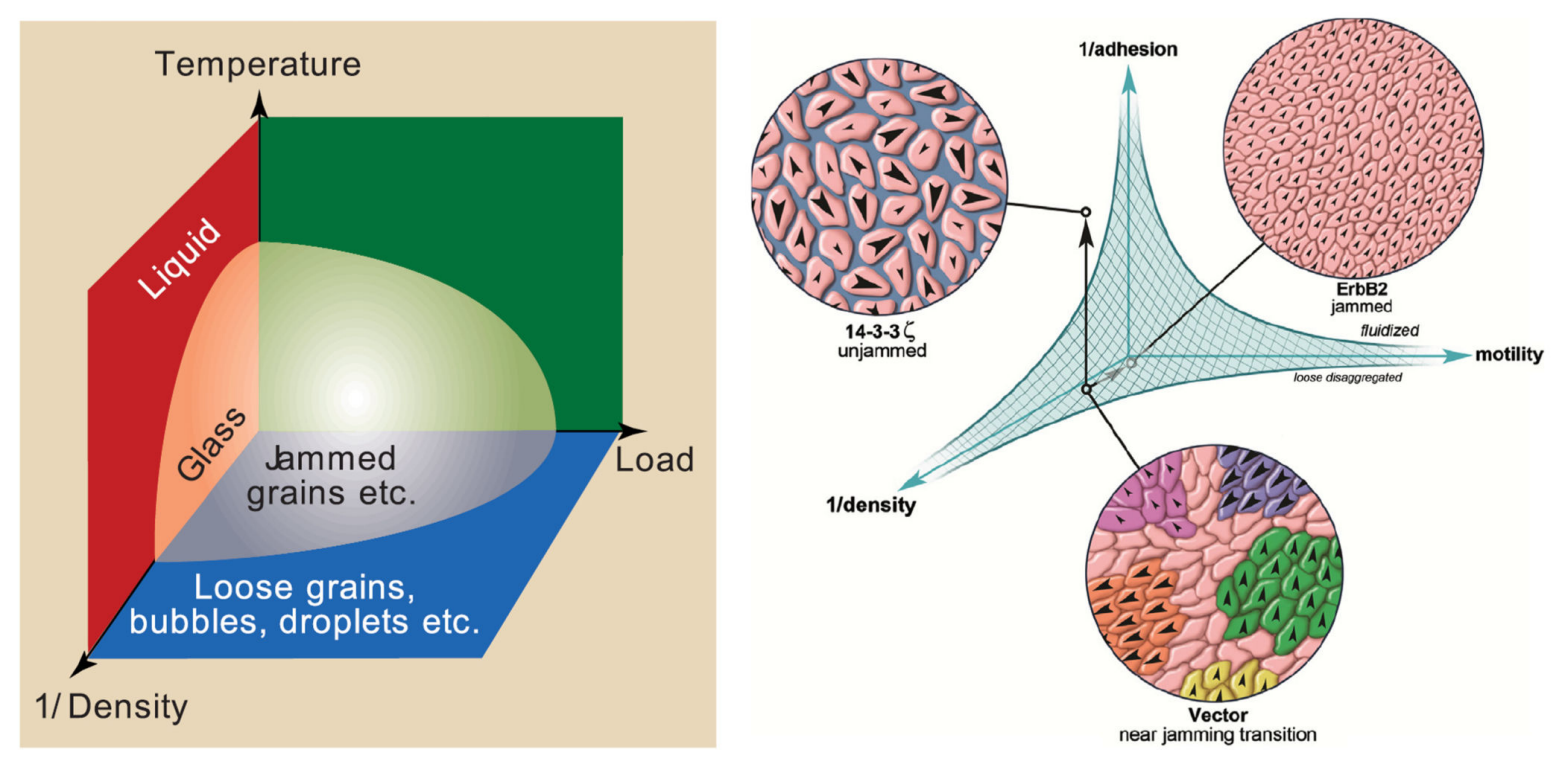
Left: jamming phase diagram proposed by [30]. Reprinted by permission from Macmillan Publishers Ltd: Nature [30], Copyright 1998. Right: hypothetical jamming phase diagram for a cellular monolayer. Reprinted from [7], Copyright 2013, with permission from Elsevier.

The intriguing similarities between jamming and glass transitions led to the proposal of a first, unifying jamming phase diagram, shown in figure 4 [30].

Here, the solid-like state is reached by decreasing the temperature, increasing the density or by decreasing the applied load. The critical parameters for a system to jam may depend on particle shape, deformability, interparticle forces and heterogeneity [29, 31, 32]. Similar to granular matter in glass forming systems, the rigidity transition is accompanied by the formation of percolation clusters [27, 33]. Jamming and glassy behaviour have received growing attention in biophysical research over the last two decades since they seem to play distinct roles in wound healing, embryonic development and cancer progression [34–37]. Jamming in tissues differs from jamming and glass transitions in non-living systems since tissues often have a constant volume fraction of one and constant temperature, thus, neither volume fraction nor thermal energy are suitable to be the dominant control parameters for the transition. More plausible triggers for a jamming transition are active motility, interaction forces, cell shape and applied stress. A phase diagram for cellular monolayers similar to that one proposed by Liu and Nagel is shown in figure 4 on the right side [7]. Here, the phase space is spanned by adhesion, motility and density.

The role of jamming in multicellular systems especially with respect to tumour development and metastasis will be subject of the next sections.

## 2 Tissues as liquids

In the last few decades, tissues have often been treated as simple or viscoelastic liquids behaving as elastic solids on short time scales and viscous fluids on long time scales. In this section, we will give an overview of experiments that have led to this picture and present the according models.

### 2.1 Experiments that have led to the broad consensus that tissues are basically liquid

**Figure 5:**
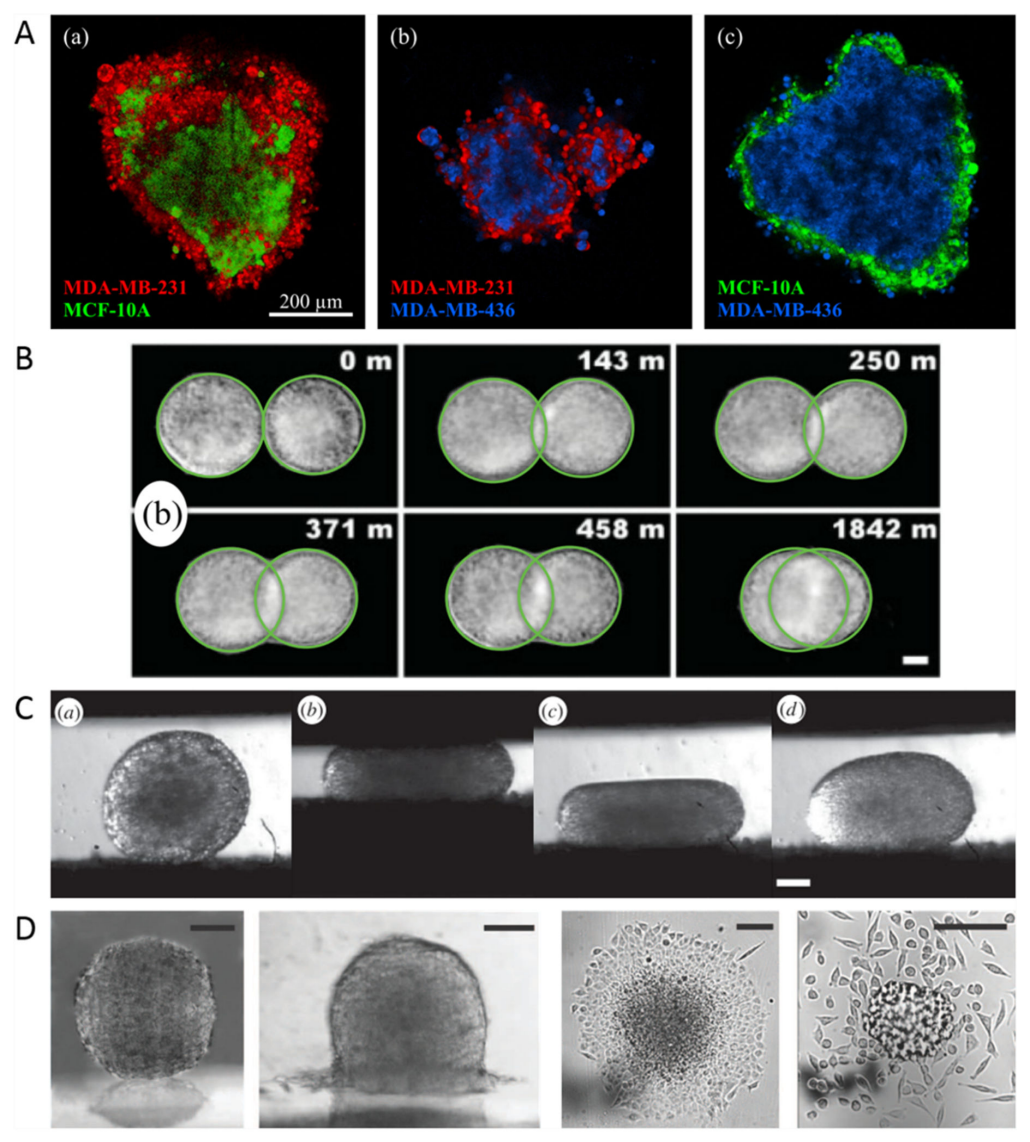
Tissues behaving as liquids. (A) Cell sorting experiments for different breast cell lines. Reproduced from [35]. © IOP Publishing Ltd and Deutsche Physikalische Gesellschaft. CC BY 3.0. (B) Fusion experiments on cushion tissue aggregates. Reprinted figure with permission from [42], Copyright 2012 by the American Physical Society. (C) Tissue compression and relaxation in a tissue surface tensiometer. Reproduced with permission from [43], © 2013 The Author(s) Published by the Royal Society. All rights reserved. (D) Partial and complete wetting of multicellular aggregates. The right image shows the transition to a 2D gas. (Far left) Reproduced from [44] with permission of The Royal Society of Chemistry. (Left) From [45]. Reprinted with permission from AAAS. (Right) Reproduced with permission from [46]. (Far right) From [45]. Reprinted with permission from AAAS.

In the early 1960s, Steinberg and coworkers published a study on *in vitro* cell sorting, i.e. the segregation of two mixed cell populations into separated clusters [38]. They showed that one cell population forms an inner sphere (e.g. heart ventricle cells), while the other one forms an outer shell (e.g. liver cells). This behaviour is analogous to the segregation of two immiscible liquids thought to be caused by differences in their surface tensions. This cell sorting demonstration has been repeated several times with different cell lines and primary cells with similar results (see figure 5(A)) [35, 39–41].

Analogous to the segregation of cells, their aggregation, rounding up and fusion (see figure 5(B)) can also be compared to liquid drops [42, 47, 48]. Mombach *et al* found that rounding up of embryo chick multicellular aggregates can be modelled as viscoelastic liquid droplets [47]. The fusion of two liquid drops is governed by their surface tension as the driving force and their viscosity as counteracting force. Particularly for embryonic tissue, the dynamics of fusion are in accordance with hydrodynamics [42, 43, 49].

The tissue surface tension (TST) can be measured with multicellular spheroids in a plate tensiometer (see figure 5(C)) [50, 51]. Most tissue types show an elastic response to tension on short time scales and a viscous response on long time scales [50, 52].

Another liquid property which can also be found in tissues is *wetting*, which describes the spreading of liquid drops on a substrate. Depending on substrate-cell interfacial tension, substrate-medium interfacial tension and cell-medium interfacial tension, non-wetting, partial wetting or complete wetting can be observed for multicellular spheroids (see figure 5(D)) [44–46]. For cell aggregates with low cohesion, complete wetting can be followed by the escape of single cells from the monolayer similar to a 2D liquid-gas transition [46]. Cells and cellular monolayers placed on non-wettable substrates (e.g. agarose gel, PEG-PLL) also form 3D aggregates by nucleation and growth, which is called dewetting [53, 54].

### 2.2 Liquid tissue models

To account for the liquid properties tissues shown in experiments, different models have been developed. As mentioned above, cell sorting is proposed to be the result of differing tissue surface tensions between the two cell populations. Steinberg and coworkers suggested that these differences arise from varying cell–cell adhesion forces mediated by cadherins [38, 41]. This hypothesis is called the *differential adhesion hypothesis* (DAH) and seems to hold true for certain cell types [41]. However, other explanations for the origin of TST have been suggested. The *differential surface contraction hypothesis* (DSCH) proposed by Harris states that TST arises from cell contractility rather than adhesion [55]. Brodland and coworkers proposed an extension of this hypothesis, the *differential interfacial tension hypothesis* (DITH), which includes both contractility and adhesion but neglects the influence of adhesion strength [56]. Experimental evidence for this hypothesis was found for gastrulating zebrafish embryos [57]. Another extension of the DAH was proposed by Manning and coworkers [35, 58, 59], which states that adhesion and cortical tension are co-regulated since cadherins are coupled to the actin cortex. Thus, down-regulation of cortex tension can lead to increased contact areas which in turn increases cohesion. This contradicts the prediction of DITH, where downregulation of cortex tension would lead to decreased surface tension. However, our own recent results on the sorting of breast cancer cell lines could not verify any of these hypotheses [35].

Another approach was presented by Basan *et al* using a continuum model for tissue dynamics [60]. Here, the steady state of the tissue is characterised by a *homeostatic pressure* which is set by division and apoptosis rates. Within this model, it has been shown that tissues behave as elastic solid in the absence of cell division and apoptosis, while otherwise they can be treated as viscoelastic fluid, with a relaxation time depending on rates of division and apoptosis [61]. The interface dynamics between two cell populations is also governed by their relative homeostatic pressures and displays two distinct regimes: a diffusive regime, where expansion is dominated by relative fluxes, and a propulsive regime, where convective flows occur due to proliferation [62].

Cells consume energy and create active forces. Consequently, some models treat them as *active liquids* in contrast to simple, passive liquids. Besides active motile forces which are included in most theoretical descriptions of cellular systems, epithelial monolayers can create active tension that scales linearly with the size of single cells [63]. The active tensile modulus depends on myosin concentration and on the specific cell type.

In contrast to the description of cell layers as active fluids, they have been proposed to behave as active granular two-dimensional matter [64]. The authors observed a solidification process in monolayers depending on the effective interparticle interaction potentials, but not primarily on density.

## 3 Local motion in tissues

To explain large scale tissue rearrangements on the cellular level, we first look at modes of cell migration. In the following, we will only give a quick overview, since reviews on this topic exist already, e.g. [65, 66].

**Figure 6:**
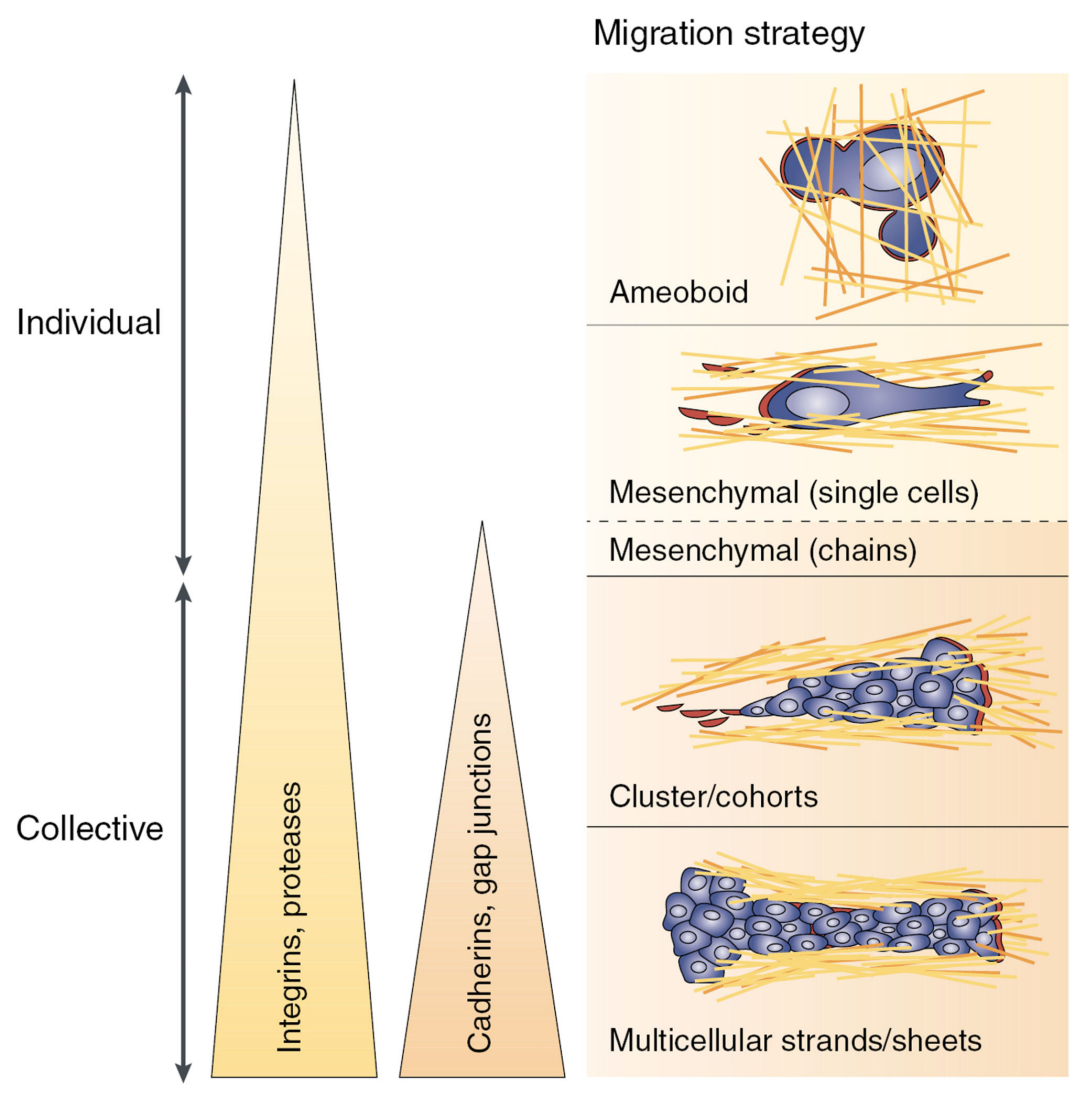
Schematic illustration of different migration modes. Adapted by permission from Macmillan Publishers Ltd: Nature Reviews Cancer [1], Copyright 2003.

Cells can move both with and without interaction with other cells. Single-cell migration, i.e. migration without cell–cell adhesion, can occur through different mechanisms, such as blebbing and filopodal amoeboid motion (figure 6, upper image), or mesenchymal motion (figure 6, second image). These migration modes can be distinguished through differences in cell-matrix interaction, cell contractility and the formation of actin stress fibres. If cells directedly move together in lines with either loose or no contact with each other, this is called multicellular streaming, which has also been observed *in vivo* [67]. If cells move together in cohesive groups, this is called collective motion (figure 6, lower two images).

Isolated cells on a 2D substrate with no obstacles are able to move under normal conditions. This changes when going to more complex 3D motility, where the microenvironment, e.g. the collagen matrix, sterically hinders cells. This mechanical blocking also happens in constricted 2D migration, such as migration through small pores where the nuclei can get stuck [6].

**Figure 7:**
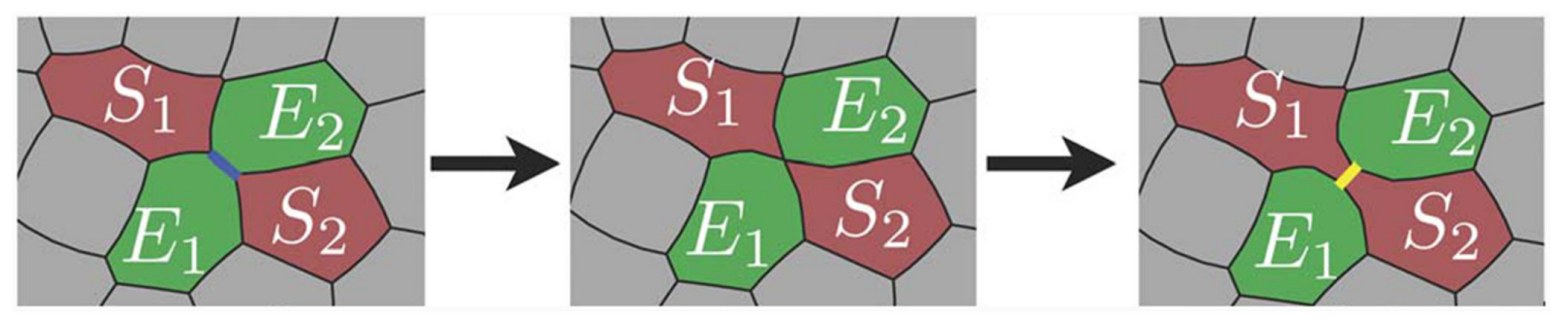
The T1 transition is a topological swap necessary for tissue rearrangement. Reproduced from [68] with permission of The Royal Society of Chemistry.

However, for tissue rearrangement in situations where cells are in direct contact with each other, such as a confluent layer or multicellular spheroids, cells have to change positions with respect to neighbouring cells in order to move. Examples for this include the compression or fusion of a multicellular aggregate on time scales where apoptosis and cell division play only minor roles. Migration in such dense tissues is different from migration in matrices. The process by which two pairs of cells swap their neighbours in a confluent layer is called T1 transition (see figure 7) [68].

Cells have to undergo shape changes during the T1 transition, hence the arrangement poses an energy barrier depending on cell–cell interfacial tension, which arises due to cortical tension, and cell–cell adhesion [69, 70]. These energy barriers can be compared to a microscopically caused yield stress, which can be overcome by the active fluctuations of the cells’ shape and by external forces [70]. If those energy barriers cannot be overcome, T1 transitions cannot take place and cells are trapped with respect to their neighbours. The material then behaves like a solid in the sense that the particles have stable equilibrium positions. David *et al* state that due to active, metabolic processes driving boundary fluctuations, tissues can also behave as a Newtonian liquid at ‘sufficiently low stress’ [69]. However, although it seems to be a reasonable assumption that active cellular systems do not jam, experimental data from several groups including our own cannot verify its veracity [49, 70, 71].

Bi *et al* found in simulations based on a vertex model for cell monolayers that the energy barrier heights of T1 transitions follow an exponential distribution, a situation which gives rise to glassy dynamics in non-active systems [68, 72, 73].

## 4 Jamming and fluid behaviour in monolayers

### 4.1 Complex phenomena in dense monolayers

Often, collective cell motion into open substrate areas has been described as ‘similar to the flow of a viscous liquid’ [74], e.g. during wound healing or the spreading of cellular aggregates on substrates. Even transitions to a 2D gas consisting of single cells separated from each other have been observed in the latter case [46]. Cellular motion in such (non-confluent) cases, where unoccupied substrate leaves room for cell motion, has also been called diffusive [75, 76].

The situation changes drastically as soon as monolayers become confluent and it is no longer a given that cells find enough room to easily bypass each other. Confluent monolayers of cells are constituted by motile, elastic and interacting particles at a 100% packing fraction. It is therefore only natural to consider that features more complex than pure fluidity emerge—glass transitions, jamming, collective motion in swirls and vortexes are only some examples known from the world of granular and colloidal matter [77].

**Figure 8:**
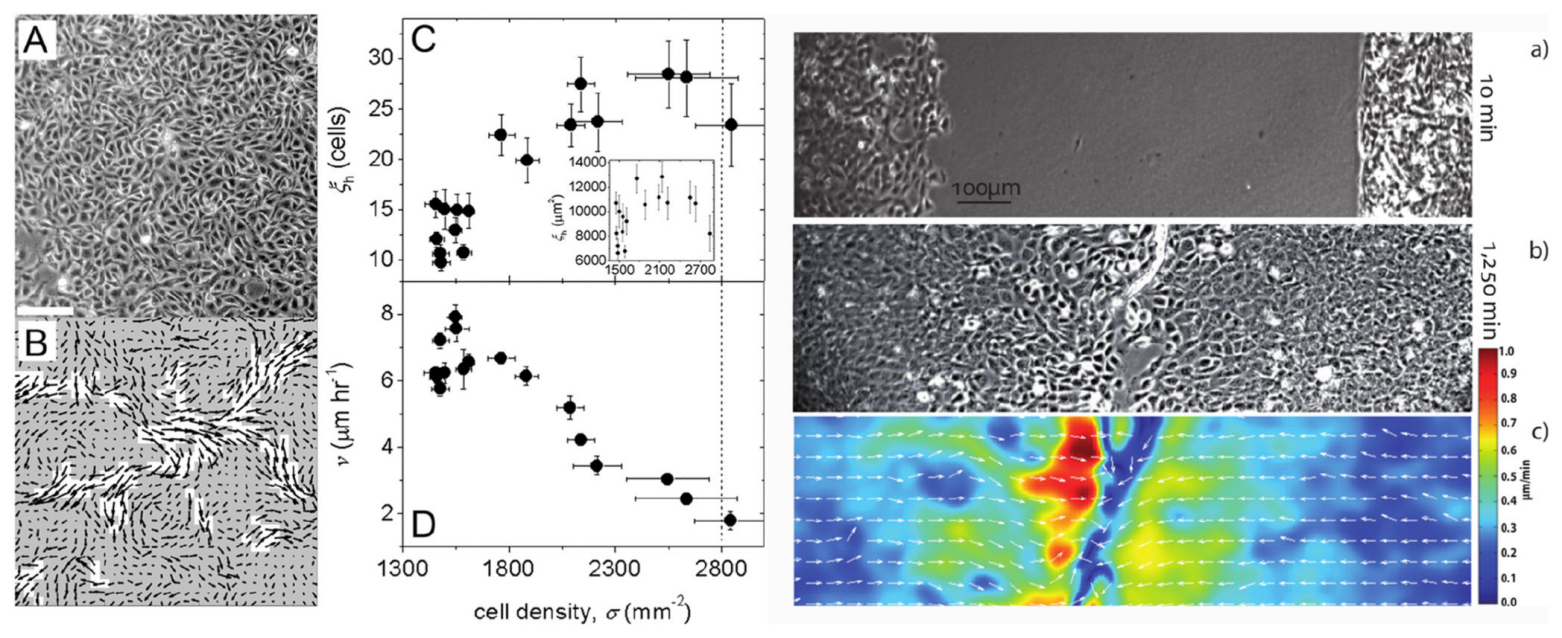
Left: with increasing density, traits of collective motility develop. Velocity autocorrelation length increases and mean speed decreases. Reproduced with permission from [34]. Right: when two identical epithelial sheets meet in wound healing, cells from both layers do not intermix but form a jammed border. Reproduced from [78]. © IOP Publishing and Deutsche Physikalische Gesellschaft. CC BY 3.0.

Angelini *et al* [34] have paved the way in 2011 by showing that a kinetic slowing down accompanied by collective motility patterns appears in confluent monolayers of MDCK epithelial cells as their number density increases. As clusters of collectively moving cells become larger, mean velocities decrease and seem to approach a state of arrest (see figures 8(A)–(D)). They have extended the notion of the dynamic structure factor by employing it to analyse time-lapse phase contrast images in a fashion similar to dynamic light scattering, demonstrating that cellular motion can be drastically different from particle diffusion in a liquid.

Do these phenomena actually occur in open setups such as wound healing? Nnetu *et al* [78] presented a study showing that the migration dynamics of expanding (epithelial) monolayers is already different from simple flow. Here, flow fields from particle image velocimetry demonstrate glass-like features. Furthermore, when two monolayers, initially separated by a wound, close the wound and clash, epithelial cells from the two opposing sides do not mix with each other, even though they are arriving at speeds much higher than those typically observed in already confluent monolayers (see figures 8(a)–(c)).

Subsequently, the route was open for further studies elaborating on the glassy behaviour of cell monolayers. Garcia *et al* [64] studied the molecular details of developing monolayers of human bronchial epithelial cells, showing that as time passes, cell–cell contacts and cell-substrate contacts maturate, leading to a higher friction between cells. The dynamical behaviours found in experiments starting from different initial cell concentrations did not collapse onto a single mastercurve when rescaled by their respective densities. Thus, they argued, it is not the number density alone which governs the transition to glassy state but rather a combination of density and increasing friction due to ageing processes. Put simply, ‘older’ monolayers begin to slow down at lower densities than ‘younger’ layers.

### 4.2 Differences in dynamics of epithelial and mesenchymal cells

These behaviours naturally raise questions as to which cellular features are responsible for the observed dynamics, and how precisely cells can modify them.

Garcia *et al* already reported that the maturation state of cell–cell contacts can catalyse or delay the critical transition [64]. Consequently, they report a different relationship between clustering and kinetic slowing down for mesenchymal fibroblasts, a cell type with less pronounced cell–cell contacts.

The same distinction between mesenchymal and epithelial cells has also been made in the aforementioned study by Nnetu *et al* [78]. When conducting a wound healing experiment with opposing cell fronts of mesenchymal NIH-3T3 fibroblasts instead of epithelial cells, they not only observe a gas-like escape of single cells at the moving cell fronts; but when the fronts meet, cells from the different sheets do not immediately jam but rather intermix.

### 4.3 Co-cultures: can healthy cells stop malignant cells?

Further elaborating on differences between cell types, Castro *et al* [37] have studied co-cultures of MCF-10A human breast epithelial cells and the MDA-MB-231 cell line, originating from a metastatic breast adenocarcinoma. While the latter cell line is of epithelial origin as well, it is assumed to have undergone EMT and can be classified as mesenchymal.

**Figure 9:**
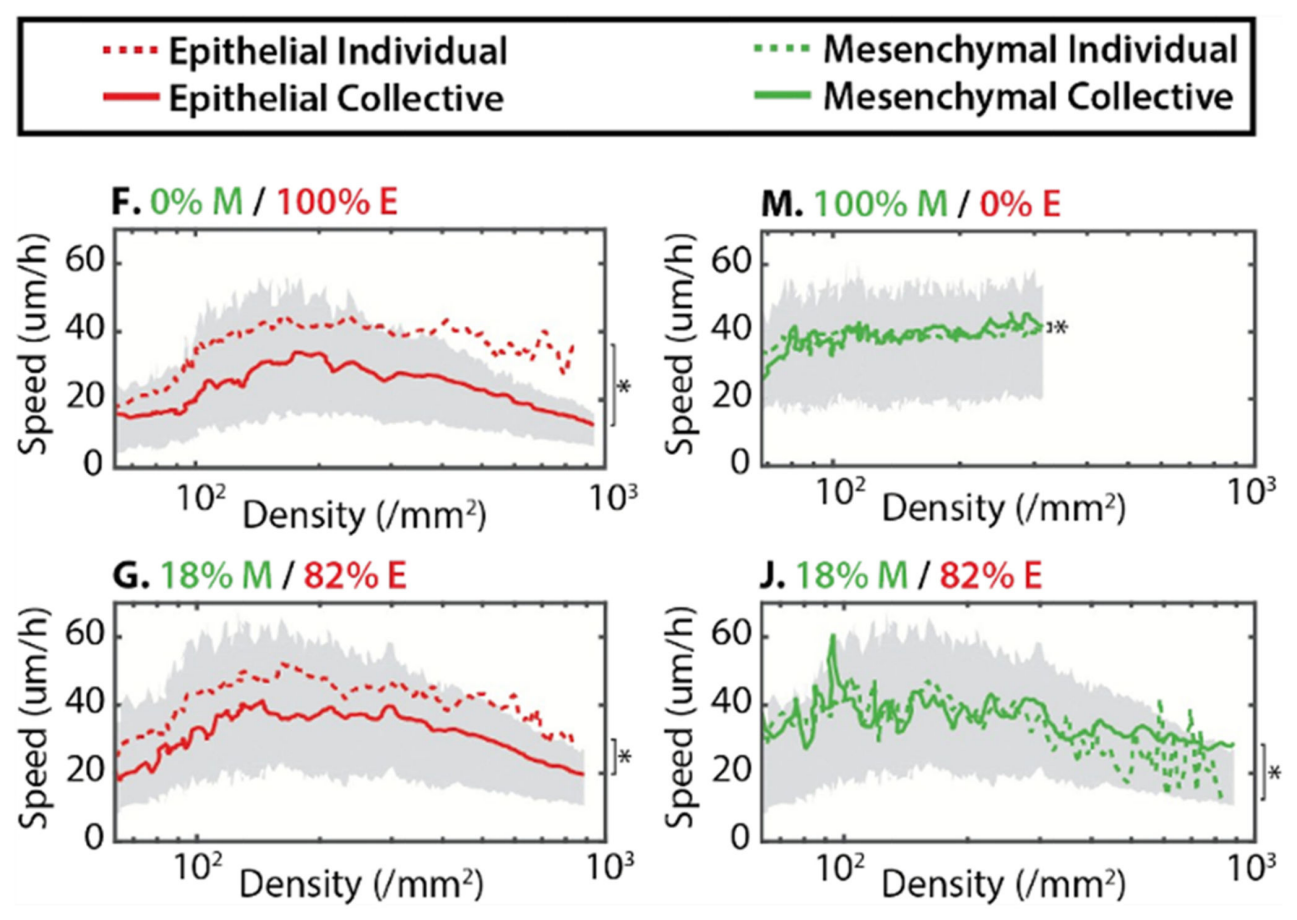
Co-cultures of epithelial (E) and mesenchymal (M) cells. (F) Epithelial cells form clusters which are slowed down at higher densities. (M) Mesenchymal cells are hardly affected by density at all. (G) In a mixture with an epithelial majority, the slowing prevails. (J) The same mixture seems to force mesenchymal cells to slow down, as they are jammed by the epithelial majority. Adapted from [37] with permission of The Royal Society of Chemistry.

Their results generally reproduce the tendencies described above, where epithelial cells slow down, cluster and jam at higher densities, while mesenchymal cell motility is hardly affected by cell density (see figure 9).

However, one observation from co-cultures, with both cell types mixed together at several different ratios, stands out: the higher the epithelial fraction, the more the MDA-MB-231 cells are slowed down at higher densities. This raises the critical question: can the epithelial cells confine and jam the malignant cell line? While mesenchymal single-cell speeds are still above the corresponding epithelial speeds, these results indicate once more that it is not the individual cell properties alone that determine a cell’s motility state, but rather that this state is dictated by the ensemble.

### 4.4 Jamming at volume fraction one and the *shape index*

The first study with primary cells from human donors where jamming is shown to play a role in a close-to-clinical context was presented by Park *et al* [36], concentrating on human bronchial epithelial cells (HBEC) from asthmatic and non-asthmatic donors.

In asthma, the airway epithelium is thought to develop mechanical instabilities leading to possible buckling and stress, therefore obstructing the airways [36]. For HBEC cells, it was found that cells from healthy donors develop glassy features in the first week after plating, while it took cells from unhealthy (asthmatic) donors longer to develop such jamming characteristics, which were less prominent even after two weeks of culture. The conjecture is thus that the lack of cell jamming in asthma either contributes or might even be necessary to the development of the mechanical unstability.

But how can we see jamming, apart from studying the dynamics, i.e. the temporal development, of the tissue sheets? Interestingly, there is a connection between static cell shapes and the dynamic behaviour of the whole sheet. While this connection had been predicted by theorists [79], it turns out that cells in a jammed tissue tend to have shapes that more closely resemble a honeycomb pattern, while unjamming requires slightly elongated cell shapes. The theory behind this will be explained in the next chapter.

**Figure 10:**
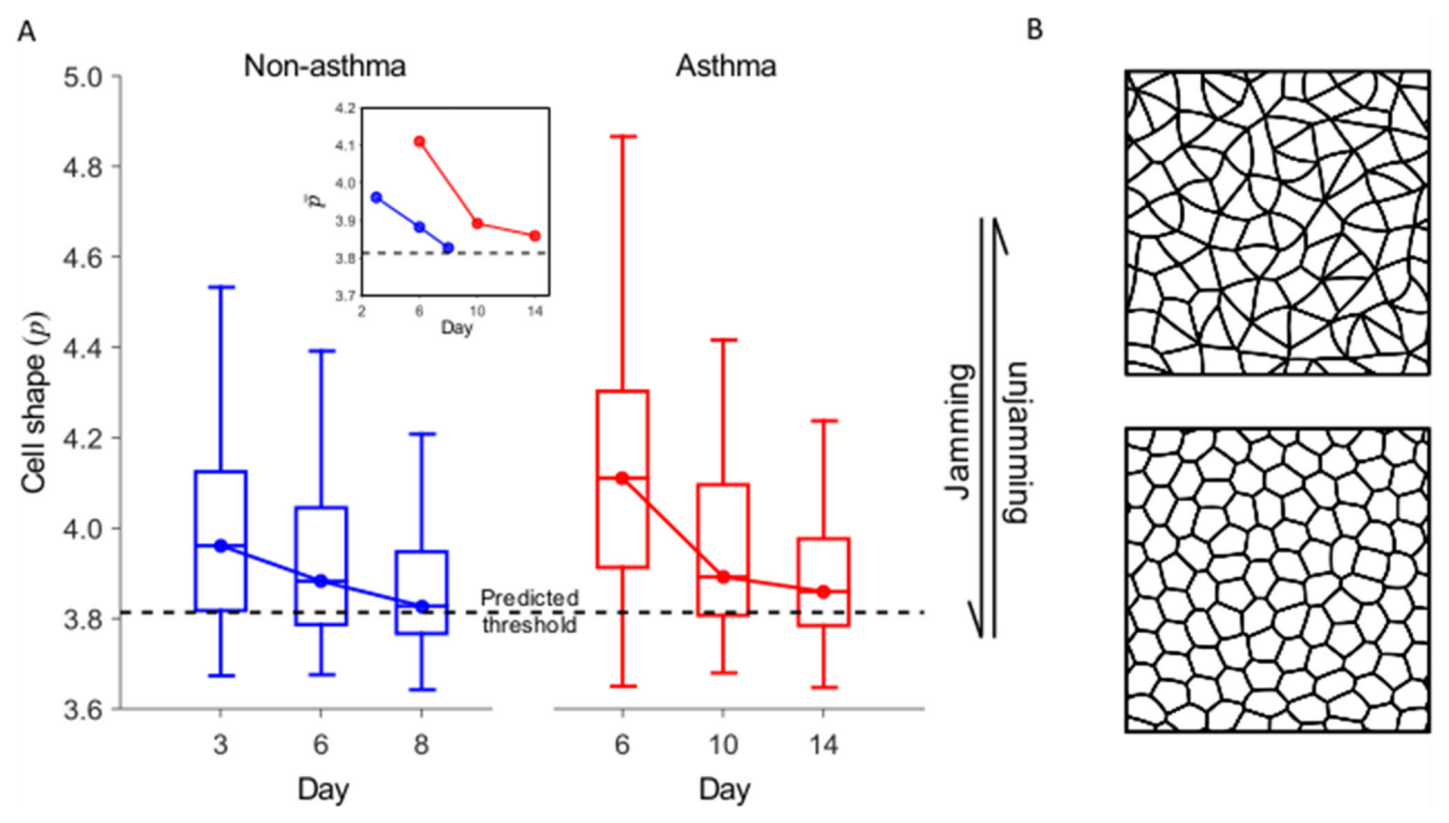
(A) The cell shape index *p*, which is introduced in chapter 5. (B) Schematics of cell shape for fluid-like (elongated) and solid-like (roundish) regime. Reprinted by permission from Macmillan Publishers Ltd: Nature Materials [36], Copyright 2015.

Indeed a cell shape change occurs concurrently to the jamming transition (see figure 10) found in the asthma study by *Park et al* [36]. This connection adds a central inquiry to an already complex picture: how does it come about that dynamic behaviours are reflected in static cell shapes, and how is this shape index defined?

## 5 Models for cell jamming

### 5.1 Vertex model and self-propelled Voronoi model for cellular jamming

A model that has received growing attention which predicts a cellular jamming transition in monolayers at volume fraction one, i.e. independent of density, is the vertex model and its extension, the self-propelled Voronoi (SPV) model [36, 37, 68, 79, 80]. In the vertex model, each cell is represented by several lattice points. The shape of each cell is governed by minimisation of the mechanical energy in the system containing *N* cells:

(1) $$E=\sum_{i=1}^{N}E_{i}=\sum_{i=1}^{N}K_{A,i}{\left(A_{i}-A_{i,0}\right)}^{2}+K_{P,i}{\left(P_{i}-P_{i,0}\right)}^{2}.$$

Here, *A_i_* and *P_i_* are the area and perimeter of each cell. *K_A,i_* and *K_P,i_* are the area and perimeter moduli, respectively. *A*_*i*,0_ and *P*_*i*,0_ are target area and target perimeter set by single-cell properties. The first term promotes volume conservation due to elastic resistance and osmotic pressure. The second term contains two effects: the active contractility of the actin cortex, which is assumed to be quadratic in perimeter, and an effective membrane tension arising from an interplay between cell–cell adhesion and cortical tension, which is linear in perimeter [68, 79, 80].

**Figure 11:**
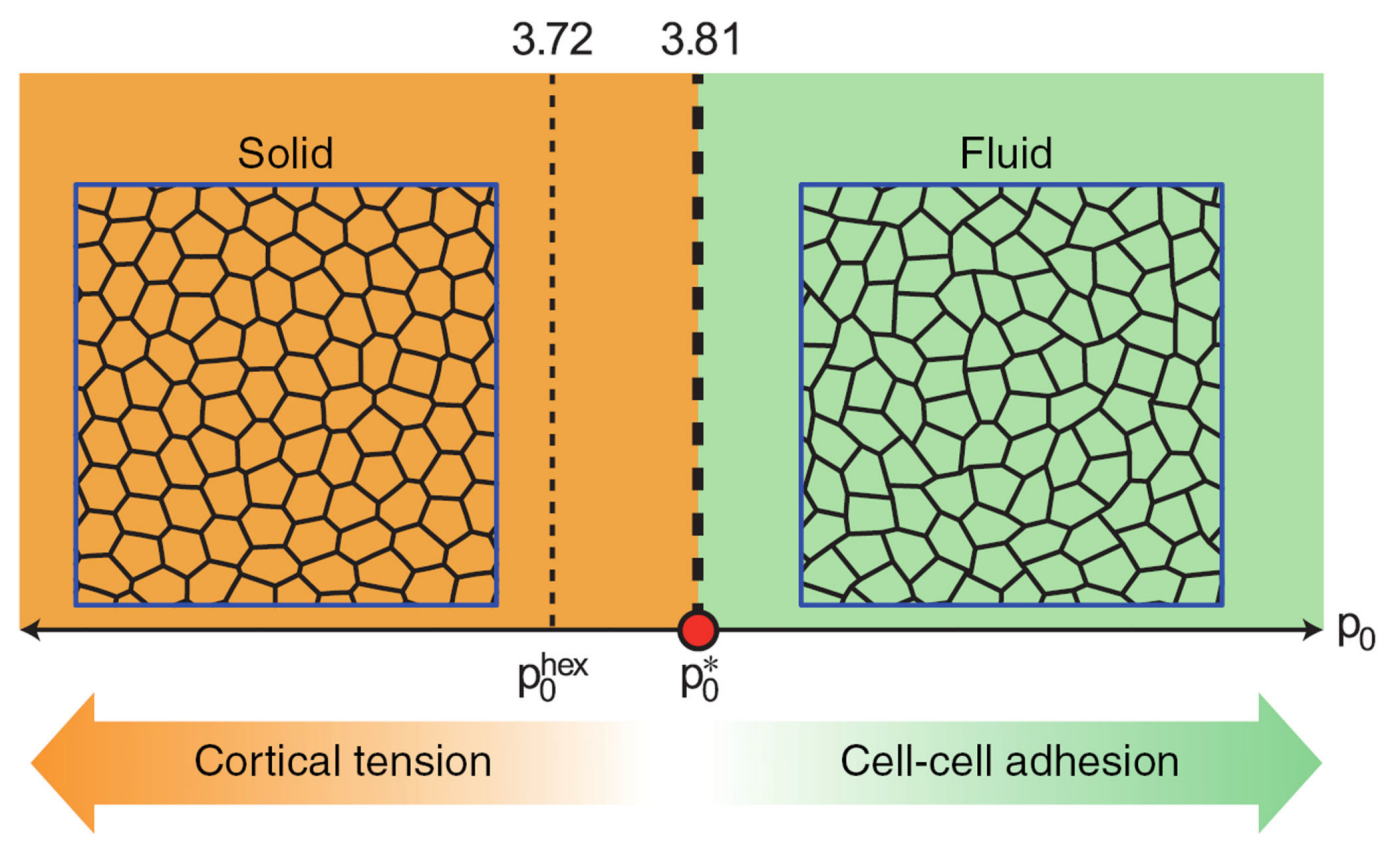
Simple phase diagram of the rigidity transition in the vertex model. The critical target shape index is $p_{0}^{*}=3.81.$ Reprinted by permission from Macmillan Publishers Ltd: Nature Physics [79], Copyright 2015.

If the system is homogeneous, the area and perimeter moduli as well as the target area and target perimeter are constant for all cells and a dimensionless target shape index $p_{0}=\frac{P_{0}}{\sqrt{A_{0}}}$ can be defined. The vertex model predicts a transition from an unjammed, fluid-like to a rigid, solid-like state if the target shape index of the system approaches $p_{0}^{*}=3.81.$ Below this value, the cells are roundish, dominated by cortical tension and the energy barriers for T1 tranistions are finite. Above this value, cells are elongated, dominated by cell–cell adhesion, and T1 energy barriers vanish (see figure 11) [79].

An extension of the vertex model is the self-propelled Voronoi (SPV) model [80], which combines the vertex model and the self-propelled particle (SPP) model. Here, each cell is identified with its centre ${\vec{r}}_{i}$ in a Voronoi tessellation of the plane. The total mechanical energy of the tissue is again given by equation (1). The trajectories of cell centres are governed by following overdamped equation of motion:

(2) $$\frac{\text{d}{\vec{r}}_{i}}{\text{d}t}=-\mu \nabla_{i}E+v_{0}{\vec{n}}_{i}.$$

Here, *μ* is the mobility, *v*_0_ is the self-propulsion velocity and ${\vec{n}}_{i}$ is the polarity vector along which the self-propulsion force is exerted.

**Figure 12:**
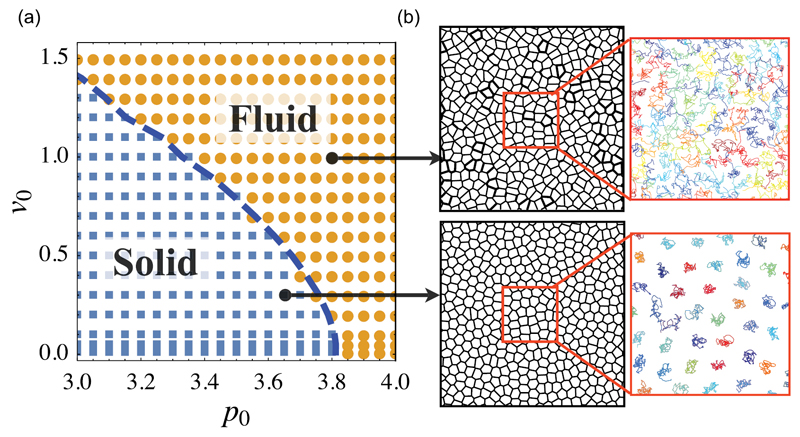
Simple phase diagram of the jamming transition in the SPV model. The critical target shape index depends on motility. Reproduced from [80]. CC BY 3.0.

Although cell shapes are acquired differently for vertex and SPV model, the latter also shows a rigidity transition at $p_{0}^{*}=3.81$ in the limit of vanishing motility. As motility increases, this critical shape index decreases as shown in figure 12.

**Figure 13:**
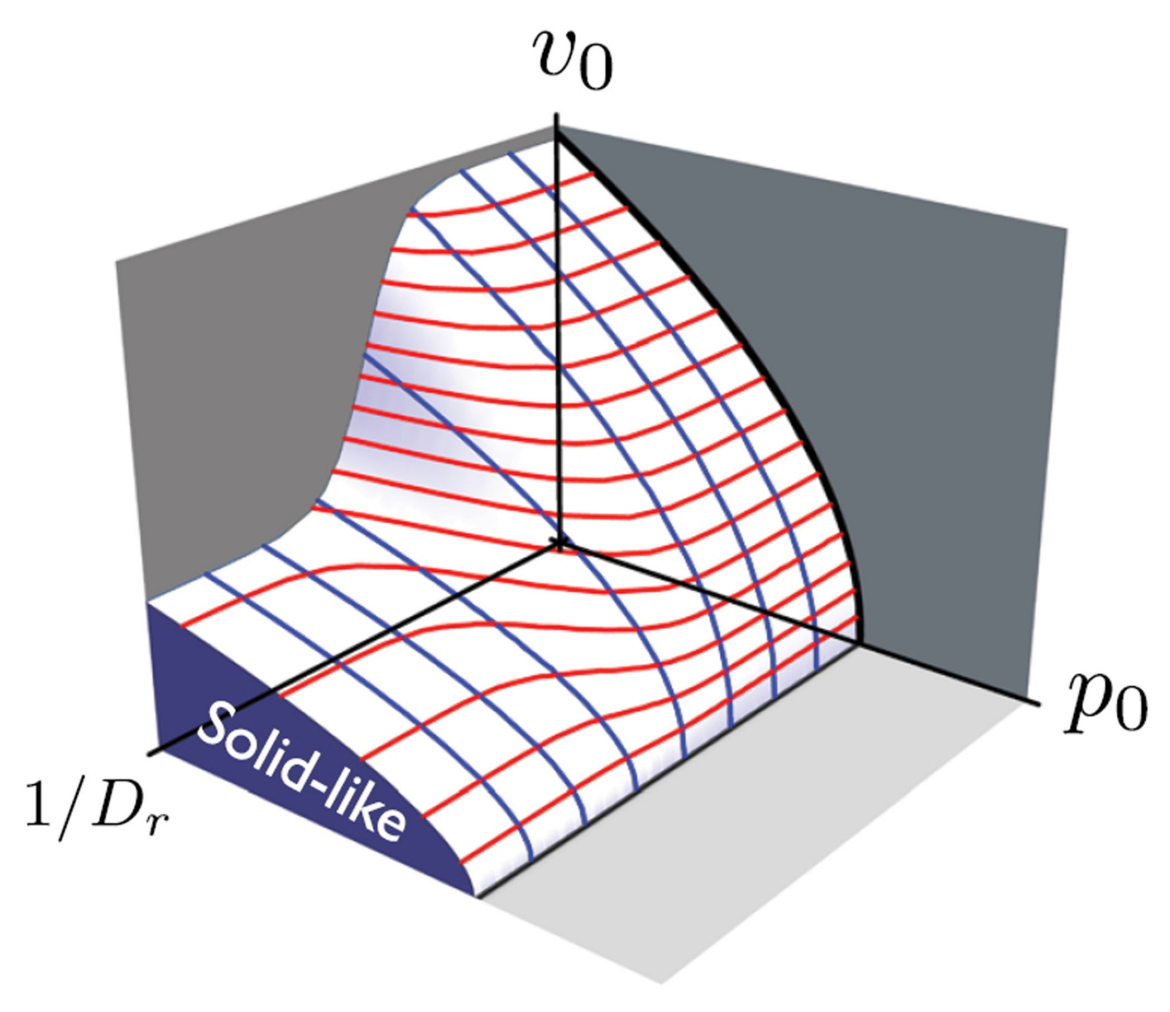
3D phase diagram for the SPV model. The jamming transition occurs in the low motility, low target shape index and low persistence time regime. Reproduced from [80]. CC BY 3.0.

In the fluid regime, cells are able to intercalate, while in the solid regime value, cells are caged and cannot change neighbours. If the polarity vector is chosen to give rise to a persistence time scale 1/*D_r_*, the phase diagram can be extended to three dimensions (see figure 13). Here, the jamming transition occurs in the regime with low motility, low target shape index and low persistence time.

In contrast to the SPV model, most SPP models predict a kinetic phase transition dependent upon not only particle density and strength of self propulsion [81–84], but also on persistence of motion [82, 85], which shifts the kinetic arrest to a higher density. For example, Henkes *et al* found two phases for self-propelled particles with soft repulsive interactions, a liquid phase at low packing fraction and high self-propulsion speed as well as a jammed phase at high packing fraction and low self-propulsion speed [81].

Until now, the number density is not included in the vertex or SPV model. However, since cells also divide in a confluent monolayer, it might be an important parameter for a jamming transition.

### 5.2 Cellular Potts model

**Figure 14:**
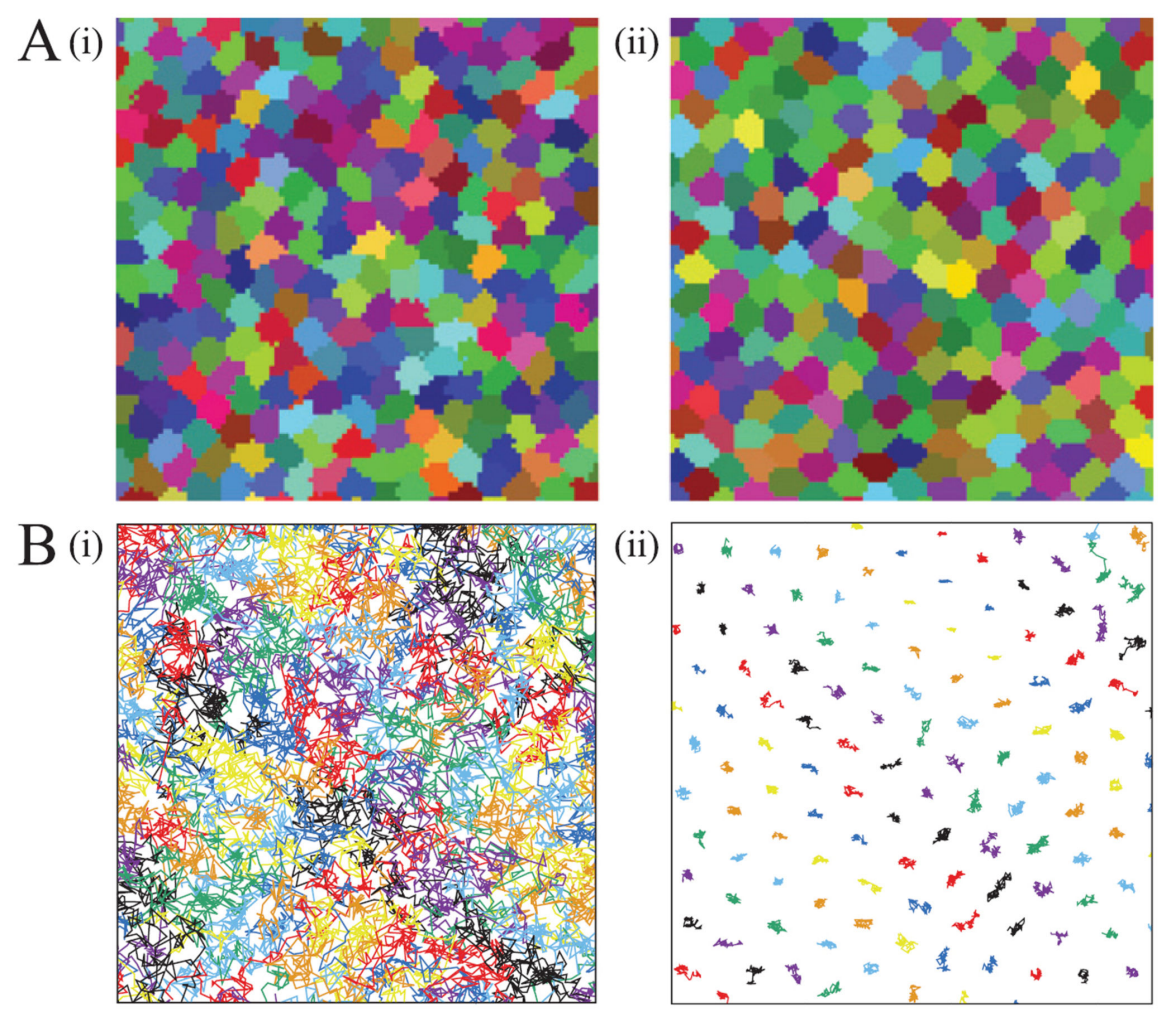
Cell shapes (A) and tracks (B) of the cellular Potts model. Figure (i) shows fluid behaviour, while (ii) shows caging. Reproduced from [86]. Copyright © EPLA, 2016. All rights reserved.

Glass transitions were not only found in the vertex and SPV model, but also in a cellular Potts model reported by Chiang *et al* [86]. A cellular Potts model is a stochastic model where cells in a monolayer are represented as regions on a lattice. The dynamic evolution calculated with a Monte Carlo algorithm is based on a Hamiltonian that includes interfacial tension, area conservation and active motility. Depending on the parameters *α* and *P*, which respectively determine interfacial energy and strength of motility, a transition from fluid-like to solid-like behaviour occurs. Cellular shapes and tracks for the two different phase states are shown in figure 14. As in the SPV model, shapes are more elongated in the fluid regime where cells can intercalate and more roundish in the solid regime, where cells are caged.

**Figure 15:**
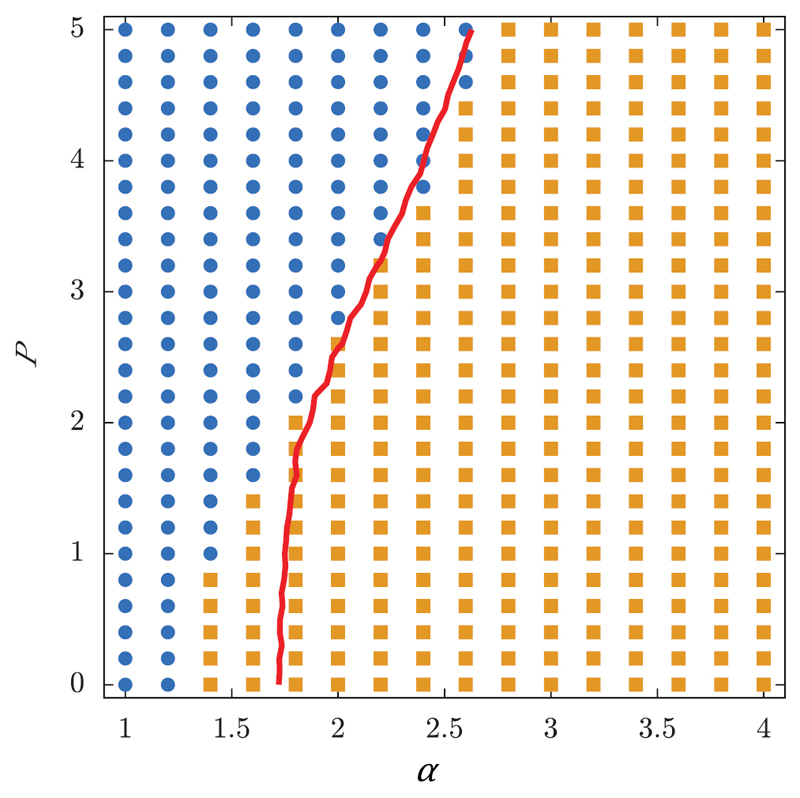
Phase diagram of the cellular Potts model. Blue dots indicate diffusive behaviour, while yellow squares indicate mean-squared displacements characteristic for sub-diffusive behaviour. The red line represents an average shape index of the cells of *p*_0_ = 4.9. Reproduced from [86]. Copyright © ELPA, 2016. All rights reserved.

The corresponding phase diagram is shown in figure 15. The blue dots indicate diffusive behaviour, while the yellow squares indicate mean-squared displacements characteristic for sub-diffusive behaviour. The red line represents an average shape index of the cells of *p*_0_ = 4.9. This critical shape index is larger compared to the critical shape index in the SPV model $(p_{0}^{*}=3.81),$ which might be due to the detailed cell shapes in the cellular Potts model. However, both models predict a glass transition which can be linked to the shape index of the cells in the monolayer.

### 5.3 Glassy cellular dynamics

To describe the behaviour of 3D zebrafish tissue explants, Schötz *et al* developed a minimal mechanical particle dynamics model containing three coarse-grained parameters which can be estimated by experiments and simulations [43]. The model includes single cell viscoelasticity, adhesion and active force generation. The equation of motion for each cell *i* is overdamped and comprises of three terms:

(3) $$0={\vec{F}}_{i}^{\text{damp}}+\sum_{\langle ij\rangle }{\vec{F}}_{ij}^{\text{int}}+\sum_{\langle ij\rangle }{\vec{F}}_{ij}^{\text{a}}.$$

The first term describes damping, the second interaction term the resistance to shape changes and adhesion and the third term an active motility towards adhesive contacts.

The non-dimensionalised equation for the centre-of-mass motion of each cell ${\vec{r}}_{i}$ can be written as

(4) $$\frac{\text{d}{\vec{r}}_{i}}{\text{d}t}=-\sum_{j}(\left(\delta_{ij}-\Gamma \right){\vec{r}}_{ij}+\sigma \xi {\vec{a}}_{ij}).$$

Here, ${\vec{r}}_{ij}$ is the unit vector between the centres of two cells, *δ_ij_* is the cell overlap, ${\vec{a}}_{ij}$ is the unit vector in the direction of the active force, *ξ* is a three-dimensional *χ*-distributed random variable with unit variance and persistence time for active forces *p_t_*. *σ* is the ratio of active force magnitude and cortical tension, and Γ is the ratio of adhesion energy and cortical tension.

The model parameters can, in principle, be calibrated using simulation and experimental data such as 3D single-cell trajectories. The predictions for bulk viscoelasticity from the model are in agreement with experimental observations, however the prediction for tissue surface tension differs, which might be due to the neglection of single cell shapes and detailed mechanical interactions.

The model can describe the observed caging effects and crossover timescales from viscous to elastic behaviour. Here, the parameters regarding magnitude and directional persistence of active forces are critical and small variations can lead to jamming or glass transitions from liquid-like to solid-like tissue behaviour. The parameters calculated for zebrafish explants are very close to this phase boundary. Similarly, low cortical tension and high cell–cell adhesion lead to solid-like behaviour, which is in contrast to predictions of both the vertex and SPV models [43].

### 5.4 Continuum descriptions

Another approach for modeling tissue dynamics utilises continuum descriptions [60, 62, 87–89]. Ranft *et al* calculated macroscopic variables such as large-scale stress distributions and flow fields of cells, both with and without cell division as well as apoptosis as sources of anisotropic stress [61]. Without division and apoptosis, the isotropic tissue behaves as elastic solid, while otherwise it behaves as viscoelastic fluid with a relaxation time set by the rates of cell division and death. If the tissue is confined in a fixed volume, it reaches a homeostatic state in which cells undergo diffusive motion.

The authors make use of dissipative particle dynamics simulations to verify the analytical results for viscosities and diffusion constants as a function of cell division rate in the homeostatic state. Cells are represented as point particles in a repulsive potential, where two cells interact repulsively on short distances and attractively on long distances. Similar to results from analytical approaches, saturating mean-squared displacements indicative of caging behaviour were found without division and apoptosis while diffusive behaviour was found if division and apoptosis are included. Other sources of noise, such as cell shape fluctuations, were not included in the model [61].

Recently, a minimal particle based model including cell–cell adhesion, cell division and apoptosis made similar predictions about the fluidisation of tissue due to cell division and death [90]. The authors produce a complex phase diagram with a gaseous, a gel-like and a self-melting liquid-like phase, where the tissue fails to show a glassy phase due to internal activity. However, glassy features were also found for embryonic tissue with normal cell division rates [43]. Thus, it seems not clear that division and apoptosis always inhibit glassy behaviour.

## 6 Falsification of pure fluidity and observation of jamming in 3D systems

### 6.1 Breakdown of DAH in 3D tumour models

**Figure 16:**
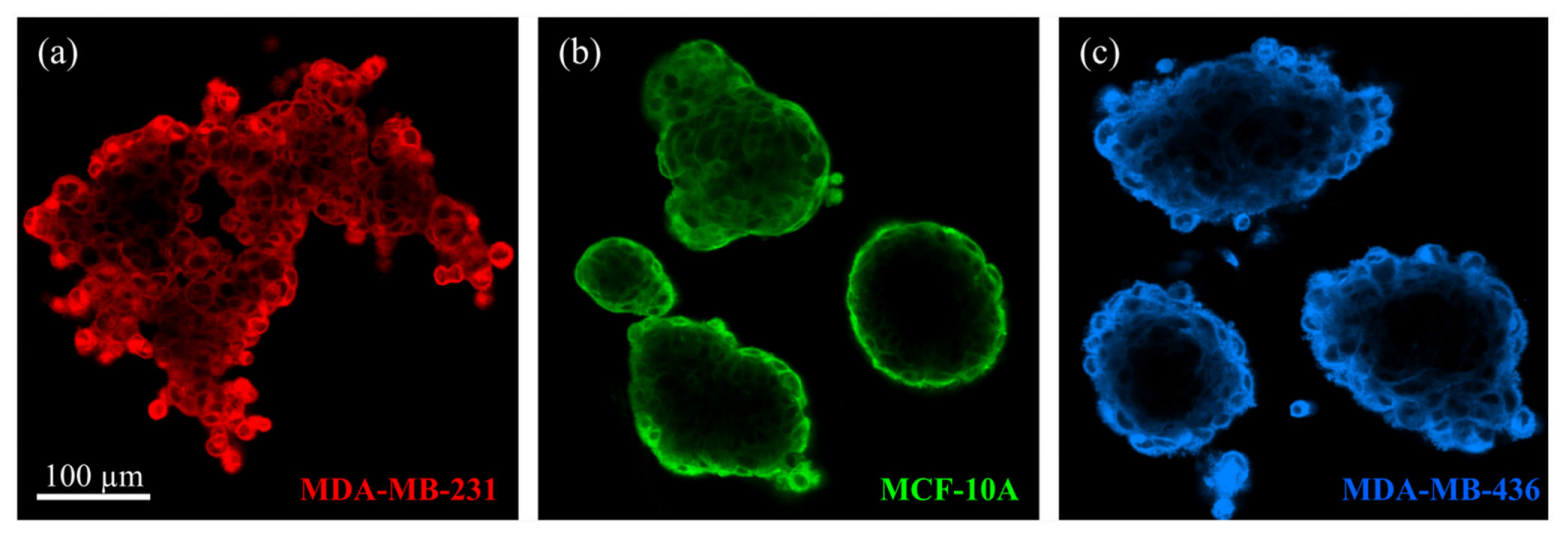
Confocal images through one plane of a 3D aggregate of metastatic MDA-MB-231 cells (a), normal MCF-10A cells (b) and cancerous MDA-MB-436 cells (c). Reproduced from [35]. © 2015 IOP Publishing Ltd and Deutsche Physikalische Gesellschaft. CC BY 3.0.

Recent studies have shown that some cell types do not only behave as fluids on long time scales in 2D, but also in 3D systems. Pawlizak *et al* [35] published a study on sorting behaviour of 3D aggregates from three different cell lines with varying metastatic potential (MCF-10A, MDA-MB-436 and MDA-MB-231, see figure 16).

**Figure 17:**
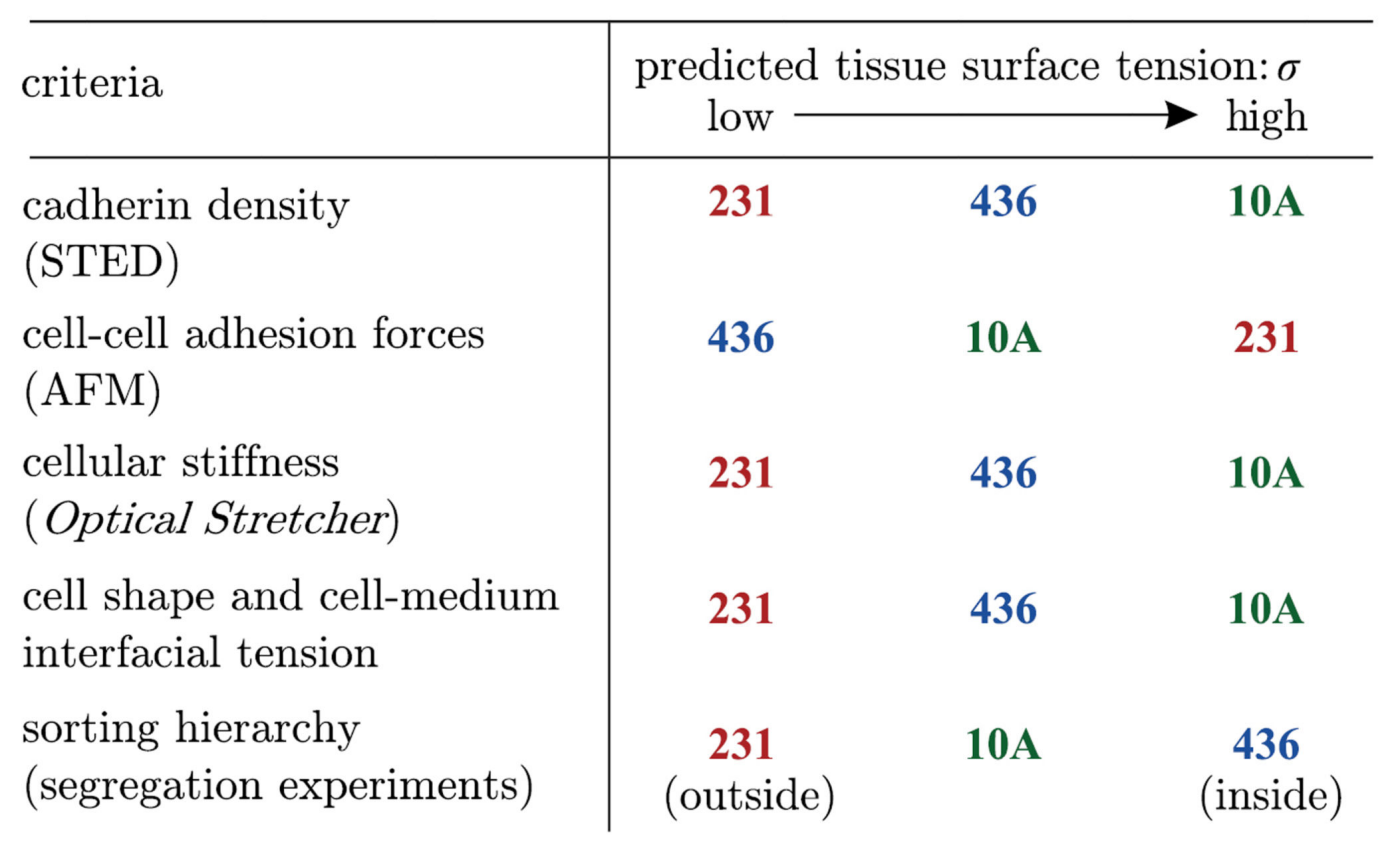
Sorting hierarchy predictions for DAH (cadherin density, cell–cella adhesion forces), DITH (cellular stiffness) and extended DAH (cell shape and cell-medium interfacial tension) compared to the observed sorting hierarchy. No prediction matches the observations. Reproduced from [35]. © 2015 IOP Publishing Ltd and Deutsche Physikalische Gesellschaft. CC BY 3.0.

Different methods were used to test DAH, DITH and extended DAH (see section 2). Cell–cell adhesion was directly measured via atomic force microscopy and indirectly by counting cadherins on the cell surface. The angles between surface cells and the aggregate were measured to test the extended DAH and whole-cell deformabilites determined by the optical stretcher were used as a measure for cell cortical tension. The resulting predictions from the different hypotheses are summarised in figure 17 and compared to the actual sorting hierarchy shown in figure 5(A).

No set of predictions based on any of these hypotheses matches the final sorted state observed in long-time imaging experiments for the cancer cell aggregates. Since all hypotheses make the assumption that tissues behave like Newtonian fluids with a defined surface tension, and even though the cell aggregates have grainy surfaces which contradicts a fluid-like surface tension, this fluidity assumption might be wrong for the tested panel of cancer cell lines [35].

### 6.2 First observations of glassy dynamics in 3D

**Figure 18:**
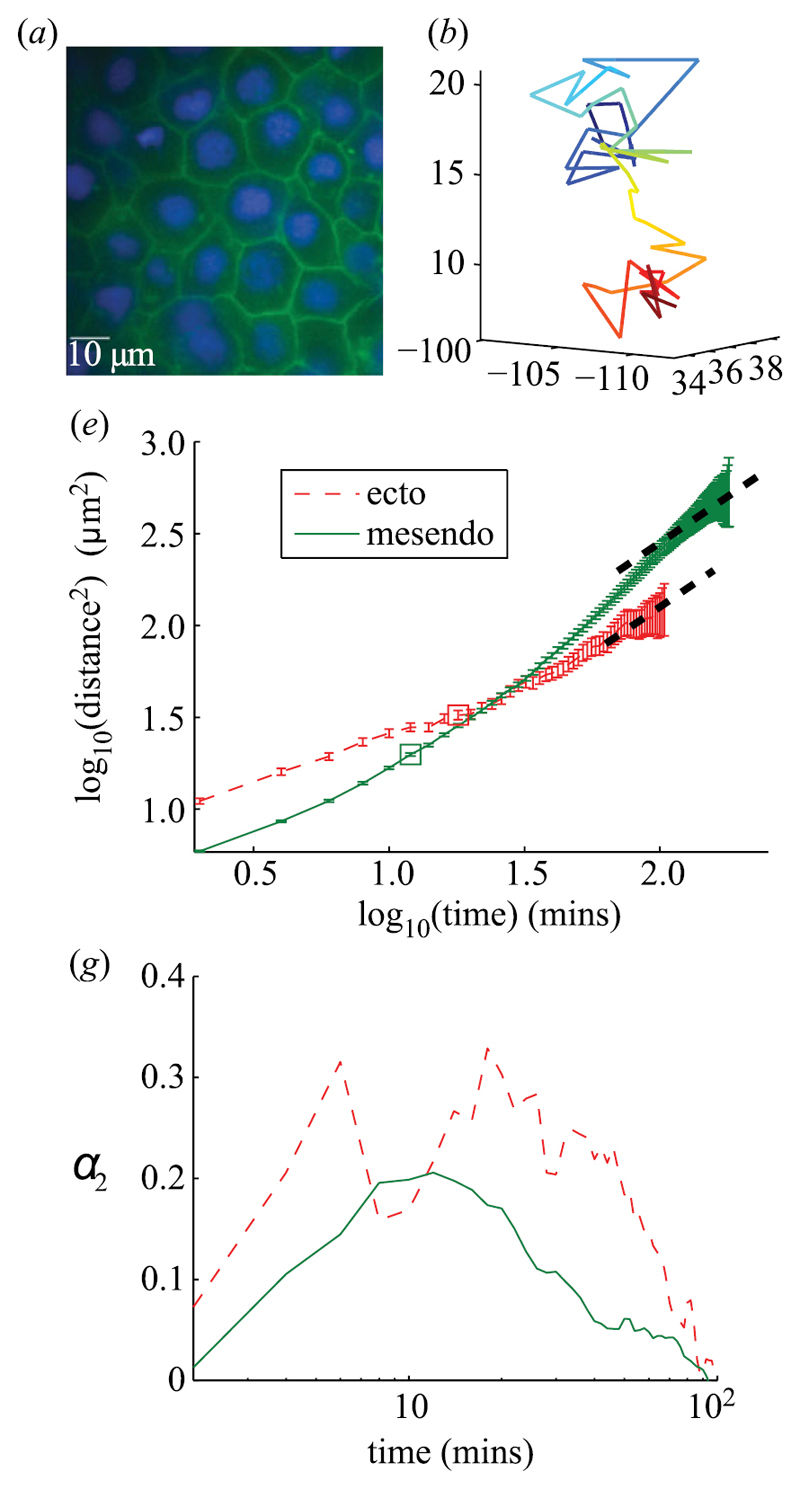
Example image of tissue (a), example track with color-coded z-coordinate (b), MSD (e) and non-Gaussian parameter *α*_2_ (g) dependent on time of ectoderm and mesendoderm Zebrafish tissue. The trajectory is reminiscent of caging, which is confirmed by the peak in the non-Gaussian parameter for intermediate time scales. The MSD shows subdiffusive behaviour (dashed lines are slope 1). Reproduced with permission from [43]. © 2013 The Author(s) Published by the Royal Society. All rights reserved.

Until now, studies on cellular jamming in 3D systems have been rare. However, Schötz *et al* recently found features of glassy dynamics in 3D tissue explants from Zebrafish embryos [43]. They analysed three-dimensional cell tracks and the macroscopic tissue response through both fusion and tissue surface tensiometer experiments. Figure 18 shows an example image of the tissue (a), an example track (b), mean-squared displacements (e) and the non-Gaussian parameter (g) as a measure for directedness of the trajectory for ectoderm and mesendoderm tissue.

The trajectory is reminiscent of caging behaviour, which is confirmed by the peak in the non-Gaussian parameter at intermediate time scales for which the energy of the cell is not sufficient to escape the cage. The MSD curves show a subdiffusive behaviour, similar to that of supercooled collids close to the glass transition. In fusion and tissue surface tensiometer experiments, Schötz *et al* found elastic behaviour on short and viscous behaviour on long time scales. Since embryonic tissues are the prime example for fluid tissues, their apparent proximity to a glass transition is an indication that tissues can exist in a jammed state [43].

### 6.3 Other hints on 3D jamming

Although not originally viewed within the context of jamming phenomena, there have beem hints pointing towards cellular jamming based on the vertex and SPV models [68, 80] in several published studies.

**Figure 19:**
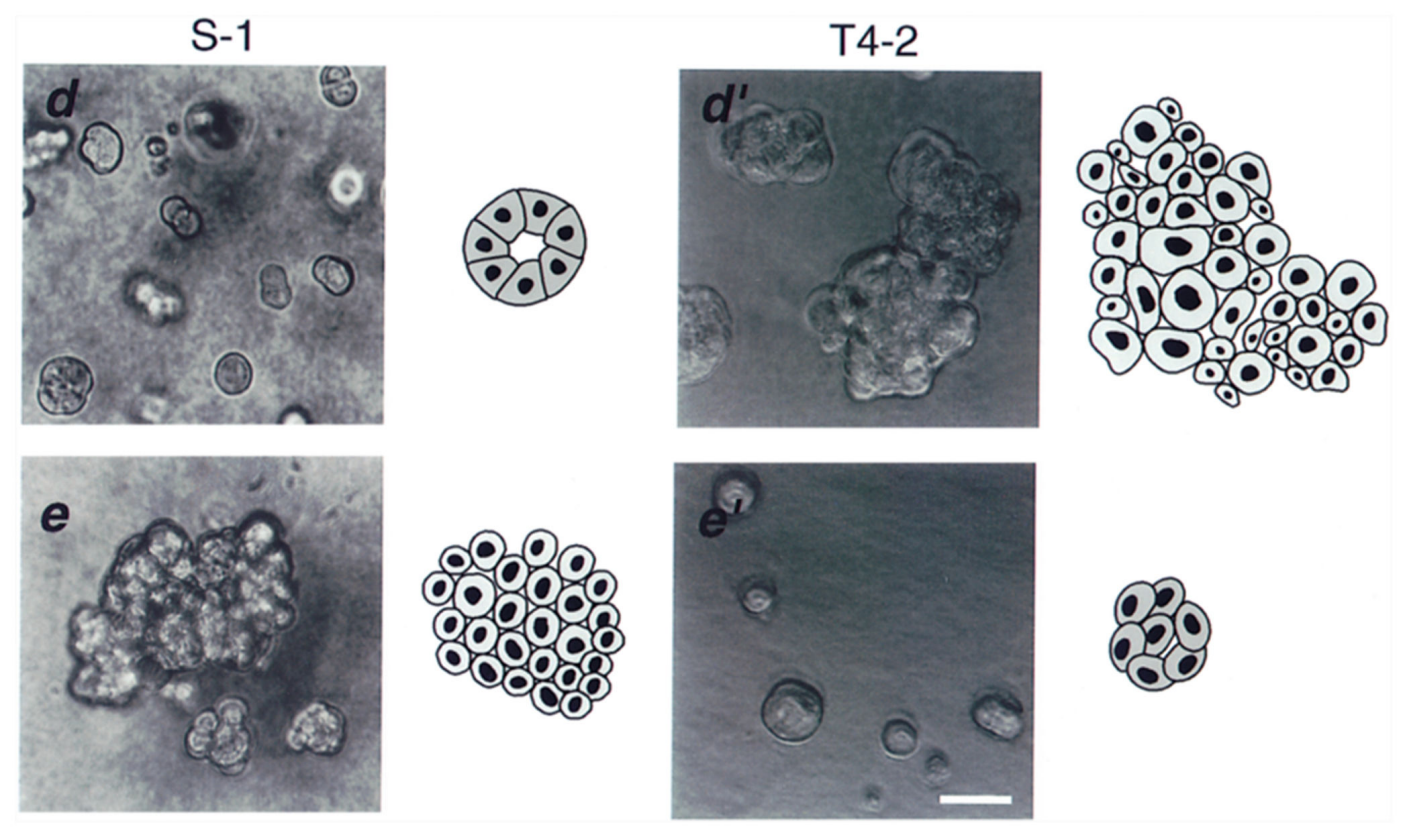
Phase contrast and schematic images of non-malignant S-1 (left) and tumorigenic T4-2 (right) cell clusters. The lower left images show S-1 cells treated with function altering *β*4 integrin antibodies, while the lower right images show T4-2 cells treated with function blocking *β*1 integrin antibodies. Reproduced with permission from [91]. © 1997 Rockefeller University Press.

For example, Weaver *et al* investigated non-malignant S-1 and malignant T4-2 breast cells embedded in 3D matrigels [91]. The cells formed aggregates which are either spherical acini in the case of S-1 or irregular clusters in case of T4-2 (see figure 19).

When S1 cells are treated with function altering *β*4 integrin antibodies, the aggregates start to grow and become disordered, similar to the T4-2 aggregates. On the other hand, when T4-2 cells are treated with function blocking *β*1 integrin antibodies, the malignant phenotype can be reverted, i.e. the clusters stop growing, a basement membrane is reassembled and a spherical shape is established [91].

This behaviour can be explained in terms of the vertex model. If integrins are blocked in T4-2 cells, adhesion in the aggregate is decreased. Thus, the target shape index is decreased and the cell system can jam, thereby forming a growth-inhibited, organised structure. For S-1 cells, where the integrin expression is increased, the analogous line of reasoning holds. Nevertheless, this hypothetical reasoning still needs rigorous experimental verification.

## 7 Clinical relevance of jamming

Cellular jamming has already been observed in experiments of clinical relevance [36] (see section 4.4). The authors used primary human bronchial epithelial cells from asthmatic and non-asthmatic donors, and observed that the non-asthmatic tissues undergo a jamming transition during maturation, while asthmatic tissues do not. Besides the dynamic characteristics, this transition is also accompanied by a change of cellular shapes from elongated shapes for unjammed tissue to rounded shapes for jammed tissue as predicted by the vertex model.

Furthermore, jamming is likely also of central importance in the growth, stasis and eventual malignant progression of tumours *in vivo*. For example, it is known that cancer cells normally divide more often that healthy cells, which are reported to be constrained by inhibited cell division after reaching confluence [92]. Since division and apoptosis are sources of stress that can fluidise a tissue [60], it is therefore plausible to argue that cancerous tissue is inherently prone to unjamming. Furthermore, inhibition of cell division in healthy tissue has been speculated to depend on cell shape rather than on contact or motion [93]. Thus, at confluence jamming might also occur due to inhibition of T1 transitions.

This implies at least two mechanisms by which unjamming transitions could be favoured in cancerours tissue: fluidisation due to increased division and increased prevalence of T1 transitions which might also arise from the lack of shape-dependent inhibition of division.

Almost 30 years ago, Mina Bissell and coworkers reported that single cancerous cells surrounded by healthy tissue with in a physiological microenvironment do not develop into neoplasia [94], suggesting that emergent tissue properties can govern single-cell behaviour. Besides the lack of signalling of the healthy, physiological microenvironment, the unjamming of tissue might also contribute to malignant progression, since the behaviour of the surroundings of the cell is qualitatively disturbed.

## 8 Conclusion and outlook

Cell jamming provides an interesting perspective on the mechanisms of tumour progression and metastasis, particularly from the point of view of physics, since it is still not known whether the jamming transition is driven by cell shape, density, or other factors. Glassy dynamics have already been observed in *in vitro* experiments, however, conclusively demonstrating that unjamming transitions can accompany the formation of metastasis in 3D tissue particularly *in vivo*, is still pending.

However, real tumours do not only consist of a collective mass of dense cells. Hence, it is also important to experimentally and theoretically investigate the general phenomena leading to and arising from single-cell jamming in the extracellular matrix or under external constrictions, and use this information to infer its influence on tumour progression. For example, jamming in the ECM might not only be influenced by jamming of the whole cell but also by nuclear blocking, which might be connected to the shape and mechanics of the nucleus.

If the formation of metastasis is indeed linked to an unjamming transition, this could be of significant importance for clinical applications. Determining the fluid and non-fluid areas in a tumour by magnet resonance elastography [8, 95] could be used as a predictive marker for cancer progression. Moreover, if cellular jamming is linked to cell shape as predicted by the SPV model, thin sections of tumours could be used to gain information on cellular dynamics from the shapes of cells within the biopsy, thus yielding a map for potential directions of metastatic invasion.
